# Complications and misdiagnoses associated with infant frenotomy: results of a healthcare professional survey

**DOI:** 10.1186/s13006-022-00481-w

**Published:** 2022-05-21

**Authors:** Mary E. O’Connor, Alison M. Gilliland, Yvonne LeFort

**Affiliations:** 1 Department of Pediatrics, Geisel School of Medicine at Dartmouth, and Childrens Hospital at Dartmouth, Hanover, NH USA; 2 Kaiser Permanente Colorado and Department of Pediatrics, University of Colorado School of Medicine, Aurora, CO USA; 3 Milford Breastfeeding Clinic and Milford Family Medical Centre, Auckland, New Zealand

**Keywords:** Ankyloglossia, Infant frenotomy, Complications, Breastfeeding

## Abstract

**Background:**

In the past 10–15 years, there has been increased concern about ankyloglossia and its effect on infant breastfeeding. This has been associated with increased performance of frenotomy. Physicians and other healthcare professionals with expertise in breastfeeding have voiced concerns about complications related to the performance of infant frenotomy. Reviews of this topic have reported no significant complications after frenotomy. Other data on complications consist of case reports.

**Methods:**

An online survey was developed by physicians with expertise in breastfeeding and e-mailed to physician and dentist members of Academy of Breastfeeding Medicine (ABM) between 11 November and 31 December 2019. It requested information from the respondents who cared for the mother/infant breastfeeding couple about their experiences personally caring for infants with complications or misdiagnoses related to referral for frenotomy or the performance of a frenotomy. Data were analyzed using chi square, Cramer’s V correlation, and binomial logistic regression.

**Results:**

Of 211 eligible respondents, 129 (61%) had cared for an infant with a complication or misdiagnosis. Two hundred and nine (209) infants were reported to have a complication and 237 had a misdiagnosis. The most common misdiagnoses reported were 101 of 237 infants (43%) with neuromuscular dysfunction and 65 of 237 (27%) with inadequate breastfeeding support. The most common complications reported were a repeat procedure considered/requested/performed 65 of 203 (32%) and oral aversion 57 of 203 (28%). Parental report of infant pain was associated with performance of a posterior frenotomy (Chi Square *p* < .003). Bleeding was associated with using scissors/scalpel vs laser/bovie/electrosurgery (Chi Square *p* = .001). Oral aversion was associated with performance of frenotomy by laser/bovie/electrosurgery vs scissors/scalpel (adjusted Odds Ratio of 4.05; 95% CI 2.07, 7.93).

**Conclusions:**

Complications and misdiagnoses are occurring after infant frenotomy. Physicians and dentists should work closely with lactation professionals to provide skilled breastfeeding support and to evaluate for other confounding problems that might impact infant breastfeeding before referral for frenotomy. Randomized controlled trials of optimized lactation support vs. frenotomy and of scissors vs laser in performance of frenotomy are needed.

**Supplementary Information:**

The online version contains supplementary material available at 10.1186/s13006-022-00481-w.

## Background

Ankyloglossia (tongue-tie) is “a condition of limited tongue mobility caused by a restrictive lingual frenulum,” that can interfere with effective and comfortable breastfeeding [1]. The prevalence of ankyloglossia ranges from 0.02% to 12 percent [2]. A recent meta-analysis showed the estimated mean prevalence of ankyloglossia was 8% (95% CI 6, 10%) in over 24,000 infants. They found higher prevalence of ankyloglossia when the diagnosis was made using a standardized assessment tool compared to visual inspection [3]. Less than 50% of infants with ankyloglossia have difficulty breastfeeding [4, 5]. Diagnosis and treatment of ankyloglossia has increased dramatically in the past two decades with studies showing significant variability in the rates of diagnosis and treatment in different areas of the countries in which it was studied [6–11]. Physicians and other healthcare professionals with expertise in breastfeeding have concerns about overdiagnosis and overtreatment [1, 12] and have questions about the diagnosis of ankyloglossia and the validity and reliability of screening tools used in the diagnosis of ankyloglossia [3, 12–14]. Frenotomy is the term for incising and releasing the tight lingual frenulum to potentially aid in tongue extension and elevation. Recent systematic reviews have concluded there are inconsistent data regarding frenotomy and breastfeeding outcomes, however it appears frenotomy may decrease maternal nipple pain and improve latch [13, 14].

One recent change in some countries is the diagnosis of posterior ankyloglossia. Posterior ankyloglossia is often used to describe a poorly defined band of tissue under the base of the tongue that limits tongue motion. However, Mills and colleagues [15, 16] found the lingual frenulum to be a dynamic fold of oral mucosa that at times includes part of the genioglossus muscle. They reported there is no direct connection between the lingual frenulum and the posterior tongue and concluded that the term “posterior tongue tie” is not anatomically correct [15, 16]. American pediatric otolaryngologists (ENTs) did not reach consensus on the definition of posterior tongue tie [1]. Other recent changes include releases of maxillary frenula and other intra-oral tissues [17–19], increased use of laser in preference to scissors for the release of oral frenula [20], and the self-referral of infants by their parents for frenotomy.

The diagnosis of ankyloglossia is challenging due to lack of uniform diagnostic criteria. Published screening tools that are available to aid in the diagnosis of ankyloglossia include the following: Koltow’s grading system (which is a classification not a screening tool), the Hazelbaker Assessment Tool for Lingual Frenulum Function (ATLFF), shortened form of the ATLFF, the Bristol Tongue Assessment Tool (BTAT), Lingual Frenulum Protocol (NTST), and Frenotomy Decision Tool for Breastfeeding Dyads [20–25]. However, none of these have been validated on large samples as predictive of a response to frenotomy. Only some parts of the ATLFF, the BTAT, and the NTST have been shown to have good interrater reliability [22–24].

Although recent systematic reviews reported no serious complications after frenotomy [13, 14], physicians and other healthcare professionals with expertise in the care of the mother/infant breastfeeding dyad have reported hearing from other physicians about infants with unreported complications after frenotomies. Underlying medical conditions can impact on the ability of the infant to breastfeed, and when a frenotomy is performed for ankyloglossia in these cases, there is concern for misdiagnosis.

## Methods

The purpose of this project was to collect and analyze information from members of the Academy of Breastfeeding Medicine (ABM) who have expertise in breastfeeding about their clinical experiences with complications and/or misdiagnoses related to infant ankyloglossia and frenotomy. We developed an on-line survey for members of ABM who are physicians and dentists regarding their experiences with ankyloglossia treatment, frenotomy, complications after frenotomy and instances where this procedure had been performed yet there were persistent breastfeeding difficulties due to an unresolved alternative diagnosis. We pilot tested the survey on members of the executive committee of the Section of Breastfeeding of the American Academy of Pediatrics and revised it. The survey was sent electronically to all 800 active members of ABM. The survey was open from 11 November to 31 December 2019. Two reminder e-mails were sent at approximately two and four weeks after the survey opening.

We collected anonymous demographic information on the respondents including: gender, age category, years in practice category, physician specialty (could list more than 1), country or region of practice, and percentage of practice time caring for the breastfeeding infant. Breastfeeding medicine as a specialty was self-defined by the respondents. We asked initial questions in this part of the survey including “Have you personally cared for any infants who have had complications after frenotomy was performed?” and “Have you cared for an infant with breastfeeding difficulties who was referred to you for a frenotomy, or after having had a frenotomy, was subsequently diagnosed with another problem that could have caused the infant’s breastfeeding difficulties?”. If yes, how many infants did you care for with a complication or misdiagnosis with fixed options of 1,2,3,4, and 5 or more. We then asked respondents to describe up to four infants and their complications or misdiagnoses. The reported frenotomy method and site were collected. Fixed options for complications were: pain reported by parent, bleeding requiring medical treatment, oral aversion/feeding refusal, scarring/retraction at the site, and repeat procedure considered/requested/performed. Fixed options for misdiagnoses were: cardiac disease, hypotonia/neuromuscular problems, infections, and cleft palate. The final option for both questions was other with a free text field. The survey included the terms “anterior frenotomy” and “posterior frenotomy” but these were not specifically defined in the survey.

This project was classified as exempt research by the Committee for Protection of Human Subjects of Dartmouth College on 11 September 2019.

The questionnaire was designed in REDCap [26, 27]. Data were analyzed in SPSS version 26 [28]. The data analysis plan was decided after data was reviewed due to lack of published data on this topic. We combined the methods of frenotomy by scissors or scalpel into one category and of laser or bovie/electrosurgery into the second for analysis, as laser/bovie/electrosurgery use heat and the scissors/scalpel do not use heat. For each specific complication, we assessed its association with the method and the location of the frenotomy. Chi Square was used for analysis of categorical variables, Cramer’s V correlation for relationships between independent variables, and Binary Logistic Regression was used when more than one variable was associated with the outcome.

## Results

There were 221 respondents of which 211 were included in the final analysis. Four respondents did not care for the mother/infant dyad, 1 survey was blank, and 5 were not physicians or dentists. We analyzed the data from all physician and dentist respondents. We did not verify ABM membership. The calculated response rate of all respondents to ABM membership was 26.4% (216/800). Demographic characteristics of the respondents are shown in Table 1.

**Table 1:** Demographic characteristics of survey respondents

Characteristic (*N* = 211)	Number (%)
**Gender**	
Female	186 (88)
Male	18 (8)
Missing	7 (3)
**Specialty**	
Pediatrics/Neonatology	119 (56)
Breastfeeding Medicine (total)^a^	102 (48)
Breastfeeding Medicine (sole specialty)	15 (7)
Family Medicine/General Practice/Internal Medicine	57 (27)
Dentist	10 (5)
Obstetrics/Gynecology	7 (3)
Surgery/Otolaryngology	1 (.5)
Other	2 (1
**Percentage of clinical time in breastfeeding**	
< 10%	24 (11)
10–25%	63 (30)
26–50%	28 (13)
51–75%	30 (14)
76–99%	25 (12)
100%	40 (19)
Missing	1 (.5)
**Years in practice**	
< 10 years	63 (30)
10–20 years	70 (33)
20–30 years	44 (21)
> 30 years	31 (15)
Missing	4 (2)
**Country/Region of practice**	
United States	130 (62)
Europe	28 (13)
Canada	15 (7)
Australia/New Zealand	14 (7)
Middle East/Asia	9 (4)
Central/South America	4 (2)
Africa	2 (1)
Missing	9 (4)

^a^87 of those reporting a specialty of breastfeeding medicine reported it as a second specialty

Seventy-eight (37%) respondents reported caring for an infant with a complication, 100/211 (47%) reported caring for an infant with a misdiagnosis, 130/211 (62%) reported caring for an infant with a complication or a misdiagnosis with 81/211 (38%) of respondents reporting not caring for an infant with a complication or a misdiagnosis. Seventy-four (56%) of respondents who classified themselves as a breastfeeding medicine specialist reported caring for infants with complications or misdiagnoses compared to 58 (44%) who did not report being a breastfeeding specialist (Chi Square *p* = 0.002). There were no differences in location of practice, years in practice, gender of provider, or clinical time caring for breastfeeding patients between those who reported caring for a patient with a complication or misdiagnosis and those who did not (Additional file 1, Table 1).

Specific information was provided for 209 of the 233 infants with a reported complication and for 237 of the 275 infants with a reported misdiagnosis. The misdiagnoses and complications are listed in Table 2. The largest category of misdiagnoses were infants with “other” and these were added to the four defined categories of low tone/hypotonia, cardiac disease, infectious disease and cleft palate where appropriate or placed in the categories of: inadequate breastfeeding support including preterm infant, abnormal suck/swallow, not ankyloglossia, genetic abnormality and other. Inadequate breastfeeding support was defined as infants who were referred for a frenotomy or did not improve after a frenotomy, but whose breastfeeding improved with lactation support alone at that point. The cleft palate category was expanded to abnormal oral/facial anatomy. Infants with abnormalities of tone were combined into one category of neuromuscular dysfunction, local or generalized.

**Table 2:** Number of misdiagnoses and complications reported on infants who were referred for or had a frenotomy performed

Misdiagnoses *N* = 237	Number (%)
Neuromuscular dysfunction, local or generalized	101 (42)
Inadequate breastfeeding support including preterm infants	65 (27)
Abnormal orofacial anatomy (cleft palate/Pierre Robin/retrognathia	28 (12)
Infectious Disease	9 (4)
Abnormal suck/swallow	7 (3)
Undiagnosed cardiac disease	6 (2)
No ankyloglossia	5 (2)
Genetic abnormality	2 (1)
Other	18 (7)
**Complications *N* = 203**	
Repeat procedure/ considered/requested/performed	65 (32)
Oral aversion/feeding refusal	57 (28)
Scarring or retraction at frenotomy site	25 (12)
Pain immediate or delayed as reported by parents	21 (10)
Bleeding requiring medical attention	20 (10)
Infection (2 abscesses, 1 mouth infection, 1 positive blood culture)	4 (2)
Other	11 (5)

Table 3 lists the specialty of the provider who performed the reported frenotomy and the reported characteristics of the procedures that were associated with the complication. The independent variables of reported site of frenotomy and method of frenotomy were significantly correlated with 88% of anterior lingual frenotomies were performed by scissors/scalpel and 91% of combined maxillary and lingual frenotomies were performed by laser/bovie/electrosurgery. Posterior frenotomies were performed by scissors/scalpel 48% of the time and 52% by laser/bovie/electrosurgery (Cramer’s V Correlation 0.613, *p* < 0.001) (Additional File 1, Table 2). Dentists performed 88% of the reported frenotomies by laser, and over 80% of the reported frenotomies performed by the pediatricians, family medicine/general practitioners and ENTs were performed by scissors/scalpel (Cramer’s V Correlation 0.528, *p* < 0.001) (Additional File 1, Table 3). Due to small numbers, we did not analyze data on maxillary lingual frenotomies alone.

**Table 3:** Reported characteristics of the provider performing the frenotomy and the method and location of the frenotomy associated with complications

Characteristic *N* = 209	Number (%)
**Provider specialty**	
Dentist	76 (36)
Pediatrician/Neonatologist	53 (25)
Otolaryngologist (ENT)	37 (18)
Family Medicine/General Practice	22 (10)
Surgeon (not ENT)	9 (4)
Midwife	3 (1)
Other	5 (2)
Unknown	4 (2)
**Frenotomy site**	
Anterior Lingual	80 (39)
Posterior Lingual	72 (35)
Maxillary and Lingual Together	45 (22)
Maxillary Only	6 (3)
Unknown	6 (3)
**Frenotomy method**	
Scissors	103 (49)
Scalpel	1 (1)
Laser	76 (36)
Bovie/Electrosurgery	11 (5)
Unknown	18 (9)

We analyzed the associations between the reports of complications and the site and method of frenotomies reported. We did not analyze data on infections due to the small numbers. There was no association between the variable scarring/retraction at frenotomy site with site or method of frenotomy. Pain as reported by the parent was significantly associated with performance of a posterior frenotomy compared to anterior, or maxillary and lingual together (*p* = 0.002, Chi Square, Table 4). Bleeding was significantly associated with the use of scissors/scalpel when compared to laser/bovie/electrosurgery (*p* = 0.001, Chi Square, Table 5). The complications of oral aversion and repeat procedure considered/requested/performed had 57 and 65 reports respectively. Both of these complications were associated the method of frenotomy and site using Chi Square analysis (Tables 4 and 5). Binary logistic regression was performed to determine whether method of frenotomy or site or both were related to these two complications. Only the use of laser/bovie/electrosurgery in performance of the reported frenotomy was associated with the occurrence of oral aversion after the procedure with an adjusted Odds Ratio (OR) 4.05 (95% CI 2.07, 7.93). Repeat procedure considered/requested/performed was associated with a prior procedure being both an anterior lingual frenotomy (adjusted OR 3.88; 95% CI 1.79, 8.40) and being performed by scissors/scalpel (adjusted OR 3.09; 95% CI 1.30, 7.34).

**Table 4:** Comparison of the reported complications with the reported location of the frenotomy *N* = 196

Complication	Anterior lingual *N* (% age)	Posterior lingual *N* (% age)	Lingual and maxillary together *N* (% age)	Statistical significance Chi square
Oral aversion				
Yes	11 (20)	24 (44)	20 (36)	*p* < 0.001*
No	69 (49)	47 (33)	25 (18)	
Scarring/retraction				
Yes	11 (46)	9 (37)	4 (17)	*p* = 0.722
No	69 (40)	62 (36)	41 (24)	
Parental report of pain				
Yes	9 (24)	23 (61)	6 (16)	*p* = 0.02*
No	71 (45)	48 (39)	39 (25)	
Bleeding				
Yes	8 (44)	9 (50)	1(6)	*p* = 0.156
No	72(40)	62 (35)	44 (25)	
Repeat procedure				
Considered/requested/performed				
Yes	43 (67)	13 (20)	8 (13)	*p* = 0.001*
No	37 (28)	58 (44)	37 (28)	

^*^Statistically significant

**Table 5:** Comparison of reported complications with the reported method of frenotomy *N* = 190

Complication	Laser/Bovie/Electrosurgery *N* (%)	Scissors/Scalpel *N* (%)	Statistical significance Chi square
Oral aversion			
Yes	38 (69)	17 (31)	*p* < 0.001*
No	48 (36)	87 (64)	
Scarring/retraction			
Yes	13 (54)	11 (46)	*p* = 0.348
No	73 (44)	93 (56)	
Parental report of pain			
Yes	18 (45)	22 (55)	*p* = 0.97
No	68 (45)	82 (55)	
Bleeding			
Yes	2 (10)	87 (90)	*p* = 0.001*
No	84 (49)	87 (51)	
Repeat procedure			
Considered/requested/performed			
Yes	10 (18)	46 (82)	*p* < 0.001
No	76 (57)	58 (43)	

*Statistically significant

## Discussion

Complications can occur after infant frenotomy, and frenotomies are being performed on infants who have undiagnosed underlying medical conditions. We found significant associations between pain as reported by the parent and posterior frenotomy, bleeding with use of scissors or scalpel for frenotomy and oral aversion with use of laser/bovie/electrosurgery. Repeat procedure considered/requested/performed was significantly associated with the prior procedure being an anterior lingual frenotomy and being performed by scissors or scalpel.

Recently, maxillary frenotomy has been recommended to improve the eversion of the upper lip and improve the latch [17, 18]. Kotlow developed a classification for assessing the maxillary frenulum [20]. Santa Maria found that the Kotlow classification had poor interrater reliability [29]. Two studies reported no correlation between the maxillary lip score by the Kotlow classification and breastfeeding outcomes in the newborn nursery [30, 31]. Mills’ Cine MR visualization of healthy breastfeeding infants, showed that the majority did not evert their upper lip while breastfeeding [32]. We did not have enough infants with maxillary frenotomies alone to evaluate for complications or misdiagnoses and in infants who had simultaneous maxillary and lingual frenotomy, one cannot determine whether one or both frenotomies were related to the complications or misdiagnoses.

Historically, frenotomy has been performed with scissors with use of the laser increasing [20]. There are no published studies directly comparing scissors/scalpel with laser or bovie/electrosurgery for performing infant frenotomy. The pediatric ENT consensus statement concluded that there is “insufficient evidence to support claims that one technique of frenotomy, such as laser, is superior to other techniques” [1]. We found that use of the laser/bovie/electrosurgery was significantly associated with oral aversion/feeding refusal as a frenotomy complication. However, use of laser/bovie/electrosurgery was associated with less reported bleeding than use of scissors/scalpel for frenotomy.

### Misdiagnoses related to infant frenotomy

The only previous report of misdiagnoses associated with infant ankyloglossia is of three infants from New Zealand. One had undiagnosed cardiac disease, one had severe poor growth and the third had undiagnosed airway obstruction [12]. The most common misdiagnoses in our study were related to infant neuromuscular issues of local or generalized hypotonia or hypertonia. The second largest group was infants with other breastfeeding problems that could be helped with good lactation support. Dixon studied a pathway of patient and provider education that reduced referral for frenotomy [33]. Caloway offered lactation support in their pediatric ENT office before performing frenotomy and found they decreased the rate of frenotomy from > 90% to 37% of infants referred after introducing lactation support. They also found that frenotomy was associated with higher maternal worries about feeding, and reduced scores on breastfeeding self- efficacy. They recommended that a functional assessment of breastfeeding is important before recommending a frenotomy [34, 35]. Bundogji performed follow-up on 220 of 343 infants one week after they performed a frenotomy in a pediatric ENT practice. Seventeen percent had stopped breastfeeding, 5% reported worse breastfeeding, 22% reported no improvement, 56% reported mild or moderate to marked improvement in breastfeeding. They concluded that healthcare professionals need to consider the many factors involved in breastfeeding before referring for frenotomy [36]. The ABM Position Statement on ankyloglossia in breastfeeding dyads supports this conclusion [37]. For the infants with a misdiagnosis, we had no information on whether the referral for frenotomy was made by a healthcare professional or parent. Our data expand on Hale’s report that misdiagnoses associated with frenotomy are occurring and that good lactation support should be provided before referral for frenotomy [12, 33, 34, 37].

### Complications related to infant frenotomy

Solis reported an infant with mucocele after a laser frenotomy and reviewed eight prior reported cases of complications after frenotomy including: two infants with Pierre Robin sequence with airway obstruction after frenotomy, four infants with infections at the frenotomy site, and two with bleeding and hypovolemia [38]. Another report describes an infant with Staphylococcus aureus infection at the frenotomy site [39]. Kim reported an infant who had severe bleeding with hypovolemic shock five days after laser frenotomy of the lingual frenulum. The infant recovered after treatment with fluids and antibiotics [40]. The Cochrane and AHRQ reviews reported no serious complications but only included studies where scissor frenotomies were performed [13, 14].

A prospective study from hospital-based pediatricians in New Zealand reported 16 infants evaluated for complications after frenotomy [12]. The most common complications noted were: poor feeding (45%), apnea/breathing difficulties (25%), pain, bleeding, or weight loss (19% each), anemia or scarring (12% each) and other (31%). Some infants had more than one complication. Seventy-five percent of the infants were hospitalized. Four infants (25%) had undergone more than one frenotomy and eight of the infants (50%) had a frenotomy in more than one location. The rate of complications/100,000 infants < one year of age varied from zero in two provinces to 85.6 (95% CI 31.4, 186) in one province with a mean rate for New Zealand of 13.9 (95% CI 7.97, 22.6). The highest rate of complications was in the province with the highest rate of frenotomy [12].

The percentage of complications of pain as reported by the parent, bleeding and scarring/retraction at the site were each < 13% of the total (Table 2) with oral aversion and repeat procedure considered/requested/performed together accounting for 60% of the complications. There is no other data for comparison. Since infants referred for frenotomy due to ankyloglossia are usually having difficulties with breastfeeding but may be drinking expressed breast milk from a bottle, developing oral aversion after the procedure is a worsening of the feeding problem. Our results show an association of oral aversion with frenotomy performed by laser/bovie/electrosurgery which has not been reported previously and expand on the complication of poor feeding after frenotomy reported by Hale [12].

The 32% complication rate of repeat procedure considered/requested/performed compares closely to the 25% of patients reported by Hale who had had more than one frenotomy performed [12], and was the most common in our study. Nelson retrospectively reviewed the rate of frenotomy revisions in their ENT practice after a change in follow-up from two weeks to one week. They found that the frenotomy revision rate decreased from 12.7% with two week follow-up to 5.2% with one week follow-up. They attributed this to more pliability of the frenotomy site that could be manipulated to improve tongue mobility [41]. They did not describe the criteria used for deciding that a frenotomy revision was needed.

The following issues could have been related to the necessity for a repeat frenotomy procedure.

1. The initial procedure was insufficient to resolve the breastfeeding issue and a further procedure was suggested /needed. This may account for the statistical association between repeat procedure being associated with previous anterior frenotomy and use of scissors/scalpel. If the previous frenotomy was a posterior lingual or maxillary and lingual together, there are few other options for further frenotomy.
2. The infant had inadequate breastfeeding support before the frenotomy (ankyloglossia was not the cause of the breastfeeding problem) or after the frenotomy. This was the second largest number of reported misdiagnoses in this report. There is no data on follow-up care after these frenotomies.
3. Other medical conditions were the cause of the breastfeeding problems. This was demonstrated in the number of misdiagnoses that occurred.

### Strengths and limitations

This paper documents the largest number of complications and misdiagnoses reported by a survey of physicians and dentists that were associated with the performance of infant frenotomy. The data is from international sites. This is the first report that associates complication with the method and site of frenotomy. This is particularly important with the increase in number of frenotomies being performed. This was a survey of physicians and dentists with an interest in care of the breastfeeding infant, so they may be more likely to care for breastfeeding infants with complications or misdiagnoses associated with frenotomy.

This project has some limitations. The low survey response rate was partially related to the fact that the first survey question asked if the respondent cared for the breastfeeding infant. If the answer was no, they were excluded by the survey. We have no data on the number of ABM members who do not care for breastfeeding infants, so our response rate may be artificially low. This was an anonymous survey and ABM members may have forwarded this to other physicians and dentists who completed the survey. This was a retrospective survey and is limited by the recall of the respondent. We did not request that respondents review the infant’s medical record. This was an on-line survey to a closed group of respondents that likely is not representative of the population of healthcare professionals caring for infants with ankyloglossia and/or treated by a frenotomy. It likely did not reach all who diagnose ankyloglossia and perform frenotomies. To address concerns that the same infant with a complication or misdiagnosis is reported by more than one respondent, we asked respondents to include only patients that they had “personally cared for”. Since the study sample included multidisciplinary respondents, it is possible there were some infants who were reported more than once. We had no information on the type of laser used for the frenotomies performed. Since this is not a population-based survey, we can’t determine rates for complications and misdiagnoses. Our conclusions are based on association and may not be cause and effect.

## Conclusions

Infant frenotomy can have associated complications and misdiagnoses, some of which are associated with location and method of frenotomy. Recommendations for avoiding complications and misdiagnoses include the following. Physicians and dentists need to work closely with lactation professionals to optimize breastfeeding support and to evaluate for other causes of breastfeeding problems before referral for or performance of frenotomy. Physicians and dentists need to be able to engage in an informed discussion and shared decision making about ankyloglossia, its effects on breastfeeding, the frenotomy procedure and its possible complications before referral for or performance of frenotomy. Physicians and dentists performing frenotomies should follow-up after the procedure or refer the infant back to their primary care physician for continued lactation support and should monitor outcomes of the infants after the procedure. Large randomized controlled trials comparing optimized lactation support vs immediate frenotomy and comparing the use of scissors vs laser for frenotomies with follow-up are needed.

## Supplementary Information

**Additional file 1: Table 1. **Demographic characteristics of survey respondents compared to the reported complications and misdiagnoses. **Table 2. **Comparison of the independent variables, reported sites of frenotomy and method of frenotomy associated with complications. **Table 3. **Comparison of the independent variables, reported specialty of provider and method of frenotomy associated with complications.

## Data Availability

The datasets generated and analyzed during this study are not publicly available because data analysis on another section of the dataset is not complete but are available from the corresponding author with reasonable request.

## Abbreviations

ABM: Academy of Breastfeeding Medicine
CI: Confidence Interval
ENT: Otolaryngologist
OR: Odds Ratio
