# Supplementary material for: Complications and misdiagnoses associated with infant frenotomy: results of a healthcare professional survey
Source: Int Breastfeed J. 2022 May 21;17:39. doi: 10.1186/s13006-022-00481-w (PMC9123702; doi:10.1186/s13006-022-00481-w)
Supplement: Supplementary file 1 — Additional file 1: Table 1. Demographic characteristics of survey respondents compared to the reported complications and misdiagnoses. Table 2. Comparison of the independent variables, reported sites of frenotomy and method of frenotomy associated with complications. Table 3. Comparison of the independent variables, reported specialty of provider and method of frenotomy associated with complications. [file 13006_2022_481_MOESM1_ESM.docx]

Additional File 1

Additional Table 1

Demographic characteristics of survey respondents compared to the reported complications and misdiagnoses

| Demographic Characteristics | Reported Complication  /Misdiagnosies  Yes (%age) | Reported Complication  /Misdiagnoses  No (%age) | Statistical Significance  Chi Square |
| --- | --- | --- | --- |
| Gender  Male  Female | 12 (67)  114 (62) | 6 (33)  70 (38) | p = .508 |
| Specialty Breastfeeding Medicine  Yes  No | 74 (73)  56 (53) | 27 (27)  50 (47) | p = .002 |
| General Specialty  Family Medicine/General Practice  Obstetrics/Gynecology  Pediatrics/Neonatology  Breastfeeding Medicine Only  Surgeon  Dentist  Other | 38 (73)  3 (38)  69 (58)  11 (85)  1 (100)  6 (60)  0 | 14 (27)  5 (62)  51 (42)  2 (15%)  0  4 (40)  1 (100) | p=.092 |
| Years in Practice  <10 years  10-20 years  21-30 years  >30 years | 36 (60)  44 (63)  27 (63)  20 (64) | 24 (40)  26 (37)  16 (37)  11 (36) | p = .975 |
| % age of clinical time spent caring for breastfeeding dyad  <10%  10-25%  26-50%  51-75%  76-99%  100% | 16 (62)  38 (61)  17 (63)  18 (62)  14 (56)  27 (71) | 10 (38)  24 (39)  10 (37)  11 (38)  11 (44)  11 (29) | p = .918 |
| Location  Africa  Asia  Australia  Canada  Europe  Middle East  New Zealand  Central South America  United States | 1 (50)  5 (71)  9 (90)  13 (87)  14 (52)  7 (78)  3 (75)  3 (75)  754 (58) | 1 (50)  2 (29)  1 (10)  2 (13)  13 (48)  2 (22)  1 (25)  1 (25)  54 (42) | p = .186 |
| Location-  Australia/Canada/Europe/New Zealand/United States  Other Countries | 113 (61)  16 (73) | 71 (39)  6 (27) | p = .30 |

Additional Table 2

Comparison of the independent variables, reported sites of frenotomy and method of frenotomy

associated with complications

| Frenotomy site | Scissors/Scalpel  N (%age) | Laser/Bovie/Electrosurgery  N (%age) | Total |
| --- | --- | --- | --- |
| Anterior Lingual | 65 (88) | 9 (12) | 74 |
| Posterior Lingual | 33 (48) | 36 (52) | 69 |
| Lingual and Maxillary Lip together | 4 (9) | 39 (91) | 43 |
| Total | 102 | 84 | 186 |

Cramer’s V .613, p<.001

Additional Table 3

Comparison of the independent variables, reported specialty of provider and method of frenotomy associated with complications

| Provider specialty | Scissor/Scalpel  N (%age) | Laser  N (%age) | Bovie/Electrosurgery  N (%age) | Total |
| --- | --- | --- | --- | --- |
| Pediatrician/Neonatologist | 42 (81) | 4 (8) | 6 (11) | 52 |
| Family Medicine/General Practice | 19 (95) | 1 (5) | 0 | 20 |
| Otolaryngologist | 26 (81) | 5 (15) | 1 (3) | 32 |
| Dentist | 9 (12) | 62 (83) | 4 (5) | 75 |
| Other Surgeon | 6 (62) | 3 (37) | 0 | 9 |
| Midwife/Unknown | 3 (75) | 1 (25) | 0 | 4 |
| Total | 105 | 76 | 11 | 192 |

Cramer’s V .528, p<.001
